# Factors associated with early mortality in haemodialysis patients undergoing coronary artery bypass surgery

**DOI:** 10.5830/CVJA-2016-066

**Published:** 2017

**Authors:** Deniz Çevirme, Taylan Adademir, Mehmet Aksüt, Kamil Cantürk Çakalağaoğlu, Alp Mete, Kaan Kırali, Tülay Örki,

**Affiliations:** Department of Cardiovascular Surgery, Kartal Koşuyolu Heart and Research Hospital, Istanbul, Turkey; Department of Cardiovascular Surgery, Kartal Koşuyolu Heart and Research Hospital, Istanbul, Turkey; Department of Cardiovascular Surgery, Kartal Koşuyolu Heart and Research Hospital, Istanbul, Turkey; Department of Cardiovascular Surgery, Kartal Koşuyolu Heart and Research Hospital, Istanbul, Turkey; Department of Cardiovascular Surgery, Kartal Koşuyolu Heart and Research Hospital, Istanbul, Turkey; Department of Cardiovascular Surgery, Kartal Koşuyolu Heart and Research Hospital, Istanbul, Turkey; Department of Anesthesiology, Kartal Koşuyolu Heart and Research Hospital, Istanbul, Turkey

**Keywords:** coronary artery bypass surgery, chronic renal failure, haemodialysis, off-pump, on-pump

## Abstract

**Introduction:**

Coronary artery bypass grafting (CABG) results in higher morbidity and mortality rates in end-stage renal disease (ESRD) patient populations than in patients with normal renal function. This study aimed to identify the early results of CABG performed on ESRD patients, and the factors that affected the mortality rates of those patients.

**Methods:**

A retrospective evaluation of our hospital database revealed 84 haemodialysis-receiving patients who underwent CABG during the years 2006 to 2012. Mortality was observed in 21 patients (group 1), and this group was compared with the remaining patients (group 2) for peri-operative parameters such as age, EuroSCORE, functional capacity, myocardial infarction, use of inotropes and completeness of revascularisation.

**Results:**

The study included 60 male (71.4%) and 24 female patients (28.6%); the participants’ mean age was 59.50 ± 9.93 years. The pre-operative additive EuroSCORE was 7.96 ± 2.88 (range: 2–18). Pre-operative functional capacity was impaired in 35.7% of the patients [New York Heart Association (NYHA) classes III–IV]. Mean age and preoperative EuroSCORE values of group 1 were significantly higher than those of group 2. Impaired functional capacity (NHYA classes III–IV) was also associated with mortality (OR: 3.333; 95% CI: 1.199–9.268).

Fifty-four patients (64.3%) underwent on-pump CABG procedures, and 30 (35.7%) underwent off-pump CABG procedures. The study found no statistically significant difference in mortality rates between these two techniques. Mortality occurred in 12 patients (22.2%) in the on-pump group and in nine (30%) in the off-pump group. Complete revascularisation was performed on 46 patients (85.2%) in the on-pump group and seven (23.3%) in the off-pump group (p < 0.001).

**Conclusion:**

Advanced age, impaired NYHA functional capacity and pre-operative hypertension were determinative for early-term surgical mortality. An on-pump surgical technique is recommended to ensure completeness of revascularisation.

## Introduction

The mortality rate of end-stage renal disease (ESRD) patients undergoing haemodialysis is high and over half of the deaths are due to cardiovascular problems.^1^,^2^ Coronary artery disease, heart failure and sudden death are the most common causes of morbidity.^3^ Coronary artery disease (CAD) is seen five to 20 times more frequently in uraemic patients than in the normal population. Because the lesions are widespread and complex, clinical prognoses worsen rapidly.^4^ Myocardial ischaemia is present because of some triggering situations in the absence of severe CAD.^5^

Heart failure occurs frequently in ESRD patients and is an independent predictor of mortality. Forty per cent of haemodialysis patients experience heart failure symptoms at the beginning of the procedure, and 25% of asymptomatic patients will develop heart failure within 3.5 years.^6^,^7^ The pre-, intra- and postoperative periods of coronary artery bypass graft (CABG) surgery must be treated more cautiously in this population. Factors affecting the morbidity and mortality rates of these patients must be well known to ensure that careful and appropriate follow up takes place. On-pump versus off-pump techniques, bleeding complications, and duration of postoperative intensive care period are among the main concerns with regard to this surgery.

The aim of this study was to identify the factors affecting the mortality rate of ESRD patients who were receiving haemodialysis and had undergone CABG procedures.

## Methods

Eighty-four chronic renal failure patients who were receiving haemodialysis and underwent CABG operations during the period 2006 to 2012 were assessed in this retrospective study. Data collection was approved by the institutional review board of our hospital and was performed in accordance with the board’s regulations.

All the patients underwent haemodialysis three times per week during the pre-operative period, and routine CABG procedures were administered to all of them. Haemodialysis was routinely performed the day before and the day after the operation.

Narcotic anaesthesia was administered intravenously to all patients. Full median sternotomy was performed on all patients; the left internal mammarian artery and the saphenous vein were used as grafts. An aortic arterial and unicaval two-stage venous cannulation was performed on patients in the on-pump CABG group; an antegrade cardioplegia cannula and a venting cannula were placed into the aortic root, and a retrograde cardioplegia cannula was placed into the coronary sinus via the right atrium.

Myocardial contraction was stopped in the diastolic phase using isothermic hyperkalaemic blood cardioplegia via an antegrade cannula, and myocardial protection was achieved using isothermic hyperkalaemic blood cardioplegia via a retrograde cannula, with the effect of systemic hypothermia. Haemodynamic monitoring was continued in the intensive care unit (ICU) and the patients underwent haemodialysis. Patients with early mortality (group 1) were compared with surviving patients (group 2) for peri-operative parameters (Table 1).

**Table 1:** Evaluation of mortality by demographic characteristics

	Mortality		
	Yes, Mean ± SD	No, Mean ± SD	p-value
Age	63.47 ± 9.89	58.17 ± 9.66	0.033*
Female, n (%)	7 (33.3)	17 (27.0)	0.577
Male, n (%)	14 (66.7)	46 (73.0)	

Student’s t-test for age; chi-squared test for gender; *p < 0.05.

## Statistical analysis

The Statistical Package for Social Sciences (SPSS) for Windows 15.0 was used to evaluate the findings of the study, and the Kolmogorov–Smirnov test was used to evaluate the coherence of the normal distribution of the study parameters. Descriptive statistical methods (standard deviation, frequency and mean) were used.

The quantitative parameters were evaluated in two parts. The Student’s t-test was used for normally distributed parameters, and the Mann–Whitney U-test was performed for non-normally distributed parameters. The Wilcoxon test was used to compare the parameters in both groups.

The chi-squared, Fisher’s exact and McNemar’s tests were used to compare qualitative parameters; p < 0.05 was accepted as significant. Univariate and multivariate analysis were used to determine independent risk factors.

## Results

Pre-operative findings were analysed according to demographic characteristics. The mean age of the patients who died was statistically significantly higher in the pre-operative evaluation (p < 0.05), as were the mean age and EuroSCORE (p < 0.05). The New York Heart Association (NYHA) functional capacity of the patients was found to be highly significantly correlated with mortality rate of patients (p < 0.01). Significantly more of the patients who died were in NYHA class III–IV (OR: 3.333; 95% CI: 1.199–9.268).

There was a statistically significant difference with regard to mortality rate between the types of surgery (p < 0.05). Significantly more patients who received emergency surgery died (OR: 10.333; 95% CI: 1.012–105.487). There was also a statistically significant difference in the incidence of hypertension (p < 0.05) between the patients who died and those who survived. The incidence of hypertension in patients who died was significantly higher than in those who survived (Table 2).

**Table 2:** Assessments for pre-operative mortality

	Mortality		
	Yes, Mean ± SD	No, Mean ± SD	p-value
EuroSCORE	9.28 ± 3.39 (9)	7.52 ± 2.58 (8)	0.040*
Ejection fraction (%)	48.33 ± 12.28 (50)	53.33 ± 10.85 (55)	0.103
NYHA class, n (%)			
1	0 (0.0)	16 (25.4)	
2	9 (42.9)	29 (46.0)	0.004**
3	10 (47.6)	18 (28.6)	
4	2 (9.5)	0 (0.0)	
Canada class, n (%)			
1	0 (0.0)	1 (1.6)	
2	11 (52.4)	30 (47.6)	0.751
3	9 (42.9)	31 (49.2)	
4	1 (4.8)	1 (1.6)	
Surgery, n (%)			
Elective	18 (85.7)	63 (100.0)	0.014*
Emergency	3 (14.3)	0 (0.0)	
Smoker, n (%)	7 (33.3)	34 (54.0)	0.101
Diabetes, n (%)	11 (52.4)	37 (58.7)	0.611
Hypercholesterolaemia, n (%)	6 (28.6)	19 (30.2)	0.890
Hypertension, n (%)	13 (61.9)	54 (85.7)	0.019*
PAD, n (%)	3 (14.3)	16 (25.4)	0.292
COPD, n (%)	7 (33.3)	18 (28.6)	0.679

Mann-Whitney U-test for euroSCORE and ejection fraction; chi-squared test for the other variables. PAD: peripheral arterial disease, COPD: chronic obstructive pulmonary disease.

The ICU length of stay and extubation time of patients who died were statistically significantly longer than those of surviving patients (p < 0.01).The incidence of pneumonia in patients who died was statistically significantly higher than in those who survived (p < 0.01; Table 3).There was also a significant difference in pre- and postoperative values of creatinine kinase-MB (CK-MB) and troponin between living and dying patients (Table 4).

**Table 3:** Assessments for intra-operative mortality

	Mortality		
	Yes, Mean ± SD	No, Mean ± SD	p-value
Transfusion (units)	2.30 ± 1.62	1.79 ± 1.24	^+^0.160
Extubation time (min)	66.90 ± 110.99	15.03 ± 8.87	^+^0.001**
ACC (min)	77.00 ± 35.37	61.24 ± 22.23	^+^0.166
TPT (min)	119.17 ± 45.30	99.90 ± 26.38	^+^0.183
Hypothermia (°C)	30.87 ± 2.19	30.98 ± 1.72	^+^0.861
Chest tube drainage (ml)	911.90 ± 580.06	652.38 ± 386.61	^+^0.067
ICU stay (day)	11.57 ± 9.08	3.93 ± 2.92	^+^0.001**
Postoperative creatinine (mg/dl)	4.56 ± 2.26	5.48 ± 2.13	^+^0.097
(μmol/l)	(403.10 ± 199.78)	(484.43 ± 188.29)	
Redo surgery, n (%)	1 (4.8)	1 (1.6)	^++^0.440
Postoperative dialysis	19 (90.5)	57 (90.5)	^++^1.000
Complete revascularisation, n (%)	12 (57.1)	41 (65.1)	^++^0.514
Pneumonia, n (%)	10 (47.6)	2 (3.2)	^++^0.001**

ACC: aortic cross clamp time, TPT: total perfusion time; ACC, TPT and hypothermia related to on-pump group (54 patients).

^+^Student’s t-test for chest tube drainage and postoperative creatinine levels; Mann–Whitney U-test for other variables; ^++^Chi-squared test and/or Fisher’s exact test; **p < 0.01.

**Table 4:** Enzyme levels

	Mortality		
	Yes, Mean ± SD	No, Mean ± SD	+p-value
CK-MB (U/I)			
Pre-op	51.90 ± 153.75	20.55 ± 16.36	0.478
Postop	135.95 ± 140.39	44.62 ± 50.56	0.006**
^++^p-value	0.003**	0.001**	
Troponin (ng/ml)			
Pre-op	27.15 ± 123.57	0.15 ± 0.38	0.379
Postop	33.14 ± 72.52	9.29 ± 10.61	0.019*
^++^p-value	0.001**	0.001**	

^+^Mann–Whitney U-test; ^++^Wilcoxon sign test; *p < 0.05; **p < 0.01.

The myocardial infarction (MI) rate in the pre-operative period was statistically significantly higher in patients who died than in surviving patients (p < 0.05; OR: 3.400; 95% CI: 1.027–11.257). The MI rate in the postoperative period was also statistically significantly higher in deceased cases than in the surviving patients (p < 0.01; OR: 8.800; 95% CI: 2.753–28.134).

There was no statistically significant difference with regard to the rate of use of intra-aortic balloon pump (IABP) in the pre-operative period (p > 0.05) between the two groups. The rate of use of IABP in the postoperative period was significantly higher in the deceased cases (p < 0.01; OR: 61.000; 95% CI: 11.422–325.788) than in the survivors.

There was no statistically significant difference with regard to mortality rate in terms of use of inotropic medicines pre-operatively between the two groups (p > 0.05). The rate of use of inotropic medicines in the postoperative period was statistically significantly higher in the patients who died than in surviving patients (p < 0.01; OR: 6.400; 95% CI: 2.181–18.784; Table 5). Patients who had undergone emergency operations and were administered inotropic medications had statistically significantly higher mortality rates.

**Table 5:** Pre-operative vs postoperative assessments in exitus patients

	Mortality		
	Yes, n (%)	No, n (%)	^+^p-value
MI, n (%)			
Pre-op	17 (81.0)	35 (55.6)	0.038*
Postop	11 (52.4)	7 (11.1)	0.001**
^++^p-value	0.070	0.001**	
CVA, n (%)			
Pre-op	2 (9.5)	7 (11.1)	0.839
Postop	2 (9.5)	1 (1.6)	0.153
^++^p-value	1.000	0.031*	
IABP, n (%)			
Pre-op	2 (9.5)	0 (0)	0.060
Postop	14 (66.7)	2 (3.2)	0.001**
^++^p-value	0.001**	0.500	
Inotrope, n (%)			
Pre-op	2 (9.5)	0 (0)	0.060
Postop	14 (66.7)	15 (23.8)	0.001**
^++^p-value	0.001**	0.001**	

^+^Chi-squared and/or Fisher’s exact test; ^++^McNemar test; *p < 0.05; **p < 0.01. MI: myocardial infarction, CVA: cerebrovascular accident, IABP: intra-aortic balloon pump.

Although complete revascularisation was not significantly related to mortality rate in both groups, the results were different when analysed with regard to the on- and off-pump group distribution (Table 6). Complete revascularisation was more frequently performed on patients in the on-pump group. The complete revascularisation rate was 23.3% in the off-pump and 85.2% in the on-pump group, and this difference was statistically significant (p < 0.01). A comparison of the mortality rates of the two groups, however, revealed no statistically significant difference (p > 0.05).

**Table 6:** Surgical technical results

	On-pump Mean ± SD	Off-pump Mean ± SD	p-value
Blood transfusion (units)	1.81 ± 1.31	2.10 ± 1.42	^+^0.225
Extubation time (min)	29.77 ± 67.07	24.80 ± 43.44	^+^0.110
ACC (min)	64.74 ± 26.18	–	–
TPT (min)	104.18 ± 32.08	–	–
Hypothermia (°C)	30.95 ± 1.81	–	–
Chest tube drainage (ml)	694.44 ± 447.91	758.33 ± 467.76	^+^0.539
ICU stay (days)	5.98 ± 5.99	5.66 ± 6.47	^+^0.712
Postoperative creatinine (mg/dl)	5.10 ± 2.23	5.52 ± 2.12	^+^0.404
(µmol/l)	(450.84 ± 197.13)	(487.97 ± 187.41)	
Redo surgery, n (%)	1 (1.9)	1 (3.3)	^++^1.000
Postoperative dialysis, n (%)	49 (90.7)	27 (90.0)	^++^0.912
Complete revascularisation, n (%)	46 (85.2)	7 (23.3)	^++^0.001**
Pneumonia, n (%)	9 (16.7)	3 (10.0)	^++^0.403
Exitus, n (%)	12 (22.2)	9 (30.0)	^++^0.430

^+^Student’s t-test for drainage and postoperative creatinine levels; Mann–Whitney U-test for other variables; ^++^Chi-squared test and/or Fisher’s exact test; **p < 0.01. ACC: aortic cross clamp time, TPT: total perfusion time.

## Discussion

The mortality rate associated with cardiovascular surgical procedures is higher for patients experiencing chronic renal failure than for patients with normal renal function.^8^,^9^ A restricted tolerance to decreased blood pressure, bleeding complications due to coagulatory problems, insufficient excretion of toxic metabolites, and sensitivity to infection may play a significant role in cases of uraemic patients.

A maturity series of 296 cases reported by Ko et al. revealed a mortality rate of 9%; the study showed that mortality occurred in patients with high NYHA functional class, left main coronary artery disease, accompanying cerebrovascular disease, or emergent surgical procedures.8 Krishnaswami et al. reported survival rates as follows: first 30 days: 91–92%; first year: 77–78%.10 Nwiloh et al. found a 20.8% mortality rate as a result of subgroup analyses in isolated CABG with ESRD patients.^11^

In Kaul and co-workers’ series of 35 cases published in 1994, the surgical mortality rate was 11.4%. They observed that congestive heart failure and a high NYHA class were determinants of mortality and that left main coronary artery disease did not affect mortality rates.^12^ Most surgeons prefer the off-pump technique because it obviates the complications of cardiopulmonary bypass (CPB) and reduces in-hospital length of stay and associated costs; however, the patient’s clinical status and risk factors limit this procedure’s applicability.

In research conducted by Chu et al., the authors examined all data and follow-up results relating to patients on whom off- and on-pump techniques were performed. The findings revealed that in-hospital mortality rates of patients in whom the on- and off-pump techniques were used were similar (3 and 3.2%, respectively). Moreover, no difference was found between the two groups with regard to postoperative stroke development in patients who were discharged.^13^ Despite this, extended hospitalisation and increased costs were observed for the off-pump group.

The physiological and anatomical features of the coronary arteries also limit the benefits of the off-pump technique. Factors that limit the success of a distal anastomosis are as follows: difficulty in reaching the coronary artery, intra-myocardial coronary artery, poor quality of the artery and arterial plaque formation, and extent of the surgeon’s experience. The results obtained from our research indicate that there was no statistically significant difference between pre-operative risk factors and postoperative parameters of patients with kidney failure who were operated on with the use of off- and on-pump techniques. The results of the mortality evaluations were likewise statistically insignificant.

Generally, complete revascularisation is the preferred approach in CABG surgery, and one of the important goals is to provide patients with good long-term NYHA functional status.^14^,^15^ Incomplete revascularisation is applied in certain cases.

In a study performed by Caputo et al., the results of incomplete and complete revascularisation with the use of off-pump techniques were compared, and a two-year follow up determined that the risk of mortality for the patients with incomplete revascularisation exhibited a statistically significant increase, compared to that of the other group.^16^ In-hospital mortality and peri-operative myocardial infarction rates were also observed to be considerably higher for the group of patients on whom incomplete revascularisation was performed. According to the data obtained from patients with chronic renal failure, incomplete revascularisation was performed on 23 patients (76.7%) in the off-pump group and four (14.8%) in the on-pump group. Complete revascularisation was found to be more prominent in the on-pump group of 46 patients (85.2%).

Two studies showed reduced platelet counts and platelet dysfunction (reduced platelet adhesiveness) due to uraemia, and an abnormal von Willebrand factor led to bleeding in chronic renal failure patients receiving haemodialysis.^17^,^18^ Nakatsu and colleagues noted the risk of haemorrhagic complications associated with heart valve surgery in ESRD patients. They found similar risk rates in bioprosthesis and mechanical heart valve patients.^19^

Prolonged extubation time and ICU stay are interrelated parameters. Pneumonia complications are due these extended times.

This cohort study had several limitations. Because it was a typical retrospective study, it suffered from lack of randomisation. The patient population was relatively small, which may have reduced our ability to detect statistically significant differences.

## Conclusion

Coronary artery bypass surgery is a highly risky approach for patients suffering from ESRD. On-pump surgery is preferred for the best surgical results, especially in elderly and high-risk patients. Peri-operative MI and an increased CK-MB level are predictive factors for mortality. However, uncontrolled hypertension and low functional capacity (NYHA) are independent determinants of mortality for this patient group.
